# Case Report: Early Valvular Repair of *Rothia mucilaginosa* Endocarditis with Intraparenchymal Hemorrhage from Septic Emboli

**DOI:** 10.5811/cpcem.41539

**Published:** 2025-07-08

**Authors:** Emma Alley, Kristy Holecko

**Affiliations:** Geisinger Medical Center, Emergency Department, Danville, Pennsylvania

**Keywords:** endocarditis, septic emboli, Rothia mucilaginosa, IV drug, case report

## Abstract

**Introduction:**

*Rothia mucilaginosa* is a rare cause of endocarditis, typically seen in intravenous (IV) drug users who use needles contaminated with saliva. However, it is rare in individuals who are immunocompetent, have no history of valvular disease, or have not undergone valvular repair. Definitive management of *R mucilaginosa* endocarditis is valvular repair, but this procedure can be delayed in the setting of intracranial hemorrhage.

**Case Report:**

We document the case of a 35-year-old male IV drug user who developed *R mucilaginosa* endocarditis, resulting in severe neurologic sequelae due to septic emboli. The patient presented to the emergency department (ED) where work-up revealed a clinical presentation consistent with endocarditis resulting in septic emboli. He was later admitted to the neurosurgical and cardiac intensive care units, where he underwent thrombectomy, monitoring of his intraparenchymal hemorrhage (IPH), and mitral valve repair. This case highlights the patient’s functional neurologic outcome following delayed mitral valve repair due to IPH.

**Conclusion:**

This case report highlights a rare form of *R mucilaginosa* endocarditis recognized in the ED, with a hospital course including thrombectomy, IPH monitoring, and mitral valve repair. The patient had progressive neurologic sequelae given delayed mitral valve repair due to concerns that procedural heparinization would worsen his IPH. Given functional decline, the patient underwent mitral valve repair on hospital day six without worsening of his IPH, demonstrating that current guidelines to delay mitral valve repair by four weeks in the setting of intracranial hemorrhage may be too strict for patients who are high risk for continued showering of septic emboli.

## INTRODUCTION

*Rothia mucilaginosa* is a rare cause of disease and endocarditis. The bacteria exist as a normal part of the flora within the human oral cavity and respiratory tracts. The pathogen is opportunistic and mostly associated with infection in the setting of immunocompromised patients or patients who have implantable devices, such as prosthetic heart valves.^1^,^2^ *Rothia mucilaginosa* infection has also been seen in intravenous (IV) drug users.^3^ In addition to endocarditis, *R mucilaginosa* has been linked with bacteremia, pneumonia, catheter-associated bloodstream infections, meningitis, peritonitis, osteomyelitis, and soft tissue infections.^1^,^2^

## CASE REPORT

The patient, a 35-year-old male with a medical history of IV drug use, active fentanyl use, cured hepatitis C post-antiviral therapy, and hypertension presented to the emergency department with left-sided facial and upper extremity numbness that began upon awakening on the day of presentation. The patient also reported right peripheral vision loss the day prior, which he thought was a complication of welding. His visual loss had not resolved by the time of presentation. He denied chest pain, shortness of breath, abdominal pain, and nausea. The patient endorsed using IV fentanyl at least three times a week and stated he reused his own needles but would never share needles.

**Image 1:**
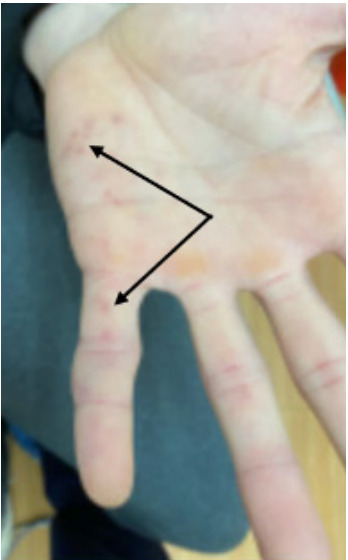
The image depicts Janeway lesions on the palm of the patient’s hand. The arrows point to the lesions.

The patient was afebrile on arrival, and all vitals were within normal limits for his age. Physical examination revealed no abnormalities in the respiratory and abdominal systems. The cardiac exam showed a 2/6 systolic murmur appreciated at the cardiac apex. Neurologic examination revealed normal sensorium and subjective left facial numbness. The remaining cranial nerves were intact, with no facial droop observed. Speech was normal, and there was no weakness in the arms or legs. Pronator drift was absent. Ophthalmologic exam was pertinent for a temporal visual field defect in the right eye with retinal exam showing hemorrhage. Dermatological exam was significant for several erythematous lesions along the hypothenar eminence of the left hand as well as on the palm of the right hand, consistent with Janeway lesions (Image 1).

Initial laboratory workup revealed mild leukocytosis of 12 x 10^3^ cells per microliter (K/μL) (reference range: 4–11 K/μL), elevated C-reactive protein at 170 milligrams per liter (mg/L) (< 5 mg/L), and erythrocyte sedimentation rate of 56 millimeters per hour (mm/hr) (<15 mm/hr). Two sets of blood cultures were obtained, eventually growing *R mucilaginosa* after admission. Computed tomography angiography (CTA) of the head and neck showed a new focal area of hypodensity in the right frontal lobe white matter and new small transcortical infarct in the left occipital lobe. Based on the CTA findings along with the physical exam and laboratory findings, the patient was admitted to the hospital for further evaluation of endocarditis with potential septic emboli. He was started on vancomycin and ceftriaxone.

**Image 2:**
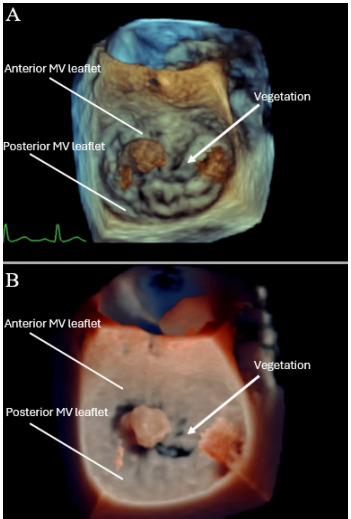
The image depicts two three-dimensional reconstructions of the transesophageal echocardiogram labeled A and B. These images show a 1.5 cm x 1.1cm homogenous, mobile mass attached to the anterior mitral valve leaflet that represents a vegetation. There is anterior leaflet perforation.

Additional imaging studies were ordered. Magnetic resonance imaging (MRI) of the brain, with and without contrast, revealed multiple parenchymal signal abnormalities, including foci of diffusion restriction throughout the bilateral cerebral hemispheres and the right superior cerebellum, consistent with multifocal infarcts. Additionally, an intraparenchymal hemorrhage (IPH) within the right frontal lobe measuring a centimeter (cm) was observed. Transthoracic echocardiogram in the ED showed evidence of a mitral valve vegetation with regurgitation. Transesophageal echo ordered inpatient showed a 1.5 cm x 1.1 cm homogenous, mobile mass attached to the anterior mitral valve leaflet that represented a vegetation (Image 2). An anterior leaflet perforation was also appreciated, with severe mitral regurgitation present. No additional vegetations were observed on the other valves.

With visualization of vegetations on the mitral valve, the patient was primarily diagnosed with mitral valve endocarditis as a result of *R mucilaginosa* infection. Given the primary diagnosis, the new multifocal infarcts throughout the cerebral hemispheres and the right superior cerebellum were attributed to septic emboli. Cardiothoracic surgery was consulted and recommended mitral valve repair, but deferred surgery for at least four weeks given the presence of IPH on MRI.

*CPC-EM Capsule*

What do we already know about this clinical entity?

Rothia mucilaginosa *is a rare form of infectious endocarditis typically seen in IV drug users*.

What makes this presentation of disease reportable?

*After Rothia mucilaginosa was identified in the ED, the patient underwent valvular repair within four weeks of intracranial hemorrhage*.

What is the major learning point?

*Patient care can be improved by rapid identification and risk/benefit analysis, although guidelines may not fit every case*.

How might this improve emergency medicine practice?

*This case highlights the need for emergency physicians to identify and advocate for patients at high risk of worsening neurologic function in the setting of continued septic emboli*.

**Image 3:**
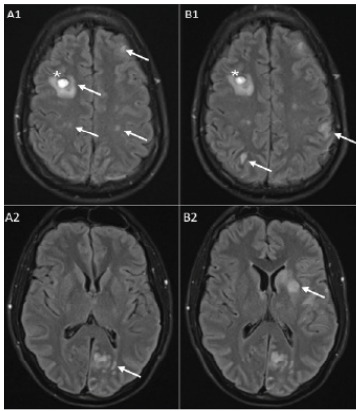
Images from a FLAIR MRI of the brain without contrast: A1 and A2 from MRI on hospital day two, show multiple parenchymal signal abnormalities throughout the cerebral hemispheres bilaterally compatible with multifocal infarcts from septic emboli (see arrows). A focus of intraparenchymal hemorrhage within the right frontal lobe measuring 1 cm is seen in A1 and redemonstrated in B1 with stable appearance (*). B1 and B2 are from MRI on hospital day 4 with new, left middle cerebral artery distribution infarcts involving the left basal ganglia, insula, corona radiata, and parietal lobe seen in B2 that were not seen prior when compared to A2. New infarcts are labeled with arrows in B1 and B2. *MRI*, magnetic resonance imaging.

Four days after admission, the patient developed sudden aphasia and right-sided arm weakness. He was taken urgently for thrombectomy after computed tomography (CT) head showed new right frontal infarct with hemorrhage. Neurosurgery found a left superior division M2 occlusion during thrombectomy. The septic emboli were successfully removed, and the left middle cerebral artery superior division of M2 was revascularized with a thrombolysis in cerebral infarction score of three. He was subsequently transferred to the neurosurgical intensive care unit (ICU) after thrombectomy, and repeat MRI was performed (Image 3). Due to the high risk of continued ischemic events from septic emboli, the decision was made to expedite the patient’s mitral valve repair, which was performed six days after presentation.

He underwent mitral valve repair with P1 triangular resection, using the semi-rigid #34 Colvin-Galloway Future Band annuloplasty ring and band (Medtronic, PLC, Minneapolis, MN). After surgery, the patient was transferred to the cardiac ICU for further care. His CT head imaging remained stable after mitral valve repair and annuloplasty. He was discharged on hospital day 15 with a peripherally inserted central catheter for continued antibiotic therapy. At the time of discharge, he still exhibited right-sided weakness and aphasia, with minimal improvement.

## DISCUSSION

Intravenous drug users are at increased risk of *Rothia* species endocarditis.^3^ *Rothia mucilaginosa* exists most commonly within the flora of the oral cavity, and it is believed that the bacteria is introduced into the blood stream of IV drug users when they lick their needles prior to injection or reuse dirty needles contaminated with saliva.^4^ Furthermore, IV drug users constitute a significant portion of endocarditis cases, often resulting in damage to native or prosthetic valves from previous episodes of endocarditis. Damaged valves and prosthetic valves provide a habitable environment for opportunistic pathogens such as *R mucilaginosa* to grow.^5^ Intravenous drug users are also at increased risk of becoming immunocompromised in the setting of HIV infections, active hepatitis infection, or cirrhosis in the setting of polysubstance abuse and co-abuse with alcohol.^6^ Our patient had no history of valvular disease but admitted to reusing dirty needles. He had a prior hepatitis C infection, which was cured with treatment before he developed endocarditis.

*Rothia mucilaginosa* endocarditis has been associated with episodes of septic emboli. In 2020 Song et al documented a case of a 65-year-old male with diabetes mellitus and alcoholic liver cirrhosis who had a history of multiple episodes of endocarditis and had a prosthetic valve placed only to subsequently develop *R mucilaginosa* endocarditis. His course was complicated by septic emboli that resulted in multiple microhemorrhages. Song and colleagues also reviewed 39 cases of intracranial complications due to *Rothia* species endocarditis and noted that 37.5% sustained intracranial hemorrhage, 25% septic emboli, and 18.7% cerebral infarction, all of which were complications in our patient. It is important to note that only 15% of the cases of *Rothia* species reviewed in their paper were *R mucilaginosa*.^7^

In our case, the patient’s neurologic sequelae were likely exacerbated by the delay in valve repair, as he continued to experience septic emboli. The Society of Thoracic Surgeons recommends delaying valvular repair in the setting of infective endocarditis by four weeks when an ICH is present, given concerns that heparinization during the procedure could worsen the ICH. However, the risk of deterioration or worsening of ICH after valvular surgery within a four-week period has not been well studied. In a similar case to ours, the authors reported valvular repair on hospital day 10 after discovery of subarachnoid hemorrhages due to septic emboli, Haddad et al describe an 80-year-old male who had a *Rothia* species aortic valve endocarditis with aortic root abscess. He underwent aortic debridement, pericardial patch placement, aortic valve repair, and mitral valve repair. No worsening of the patient’s subarachnoid hemorrhages or neurologic function was reported after repair.^9^

Yoshioka et al performed a retrospective study to investigate stratified risk in patients who had preoperative ICH prior to valvular surgery in the setting of infective endocarditis. They examined 30 patients with valvular repair ranging from within seven days of ICH onset up to >29 days of onset. They found that none of the 30 patients had worsening of their ICH after the procedure. Two patients developed new asymptomatic ICH after the procedure, one who was eight days out and the second 81 days out from onset of their preoperative ICH.^10^ Our patient ultimately underwent valvular repair approximately six days after the onset of IPH, without any complications of worsening ICH.

## CONCLUSION

The findings from Yoshioka et al and the Haddad et al case report, along with our case, present an interesting contrast to the recommendations provided by the Society of Thoracic Surgeons. While *R mucilaginosa* is susceptible to beta-lactams and vancomycin, studies have shown that early valvular repair has been linked with improved mortality outcomes in patients with infective endocarditis over treatment with antibiotics alone.^9^–^11^ Understanding that functional outcomes for infective endocarditis are improved by early valvular repair highlights two important needs for better outcomes of patients with intracranial hemorrhage from septic emboli: 1) early identification of endocarditis, which can occur within the ED; a thorough history to identify risk factors, an in-depth physical exam, and use of diagnostic imaging, such as CT, MRI, or ultrasound to identify infective endocarditis and septic emboli before admission, as was seen in our case; and 2) further investigation into whether ICH from septic emboli should be listed as a relative vs absolute contraindication to valvular repair within four weeks, as early repair may help prevent the worsening of neurologic sequelae from continued septic emboli as we reported.
